# Oxytocin receptor gene and parental bonding modulate prefrontal responses to cries: a NIRS Study

**DOI:** 10.1038/s41598-020-65582-0

**Published:** 2020-05-22

**Authors:** Ilaria Cataldo, Michelle Jin-Yee Neoh, Wei Fang Chew, Jia Nee Foo, Bruno Lepri, Gianluca Esposito

**Affiliations:** 1 Affiliative Behavior and Physiology Lab, Department of Psychology and Cognitive Science, University of Trento, Rovereto, TN Italy; 2 Mobile and Social Computing Lab, Bruno Kessler Foundation, Trento, Italy; 3 Social and Affective Neuroscience Lab, Psychology Program, School of Social Sciences, Nanyang Technological University, Singapore, Singapore; 4 Lee Kong Chian School of Medicine, Nanyang Technological University, Singapore, Singapore; 5 Human Genetics, Genome Institute of Singapore, Singapore, Singapore

**Keywords:** Behavioural genetics, Social behaviour, Human behaviour

## Abstract

The ability to interpret and regulate emotions relies on experiences of emotional socialization, obtained firstly through the interaction with the parents, and on genetic features that affect how individuals take on social situations. Evidence from the genetic field states that specific allelic variations of the oxytocin receptor gene polymorphisms regulate physiological modulation of human behavior, especially concerning responses to social cues and affiliative behaviors. Starting from this gene-by-environment interaction frame, we assessed 102 young adults for OXTr rs53576 and rs2254298, recalled parental bonding (using the Parental Bonding Instrument), and recorded participants’ neural responses to social stressors using Near InfraRed Spectroscopy (NIRS). The results highlight that higher genetic susceptibility (G/G homozygous) to familiar context and positive early life interactions modulate more optimal neural responses to general social cues, in terms of promptness to action. With regards to the dimensions of parental bonding, we found lateralized effects, with greater activation in the right prefrontal cortex for Care subscales, and on the left side of the prefrontal cortex for Overprotection. Results provide evidence to understand the neurological mechanisms behind the negative impact of poor parenting practices on the child.

## Introduction

Major theories in developmental psychology and mental health fields have proved that child’s development is widely influenced by initial experiences with caregivers^1^. In particular, parental bonding affects different aspects of an individual’s life, such as academic performance, reaction to stress loads, and risks of psychopathology^2–5^. Additionally, the ability of children in interpreting, regulating, and communicating emotions relies on experiences of emotional socialization obtained through active interactions with parents and other close people^6^. In comparison to adverse parenting practices (e.g., parental hostility), positive ones (e.g., maternal sensitivity) are associated with better emotional regulation abilities^7^, which persist in shaping social interactions. This is because proper emotional regulation facilitates the development of empathy, rather than anxiety, in response to the distress of a social other^6^. Notably, early childhood experiences with the caregiver also formulate an individual’s inclination towards perceiving the self and the social information^8–10^. Caregiving experiences could also inhibit or motivate our interactions in situations without perceived security^11^. In sum, previous findings clearly illustrate that parenting influences a child’s social-emotional development, and that affects how individuals deal with social situations. Social information processing theory (SIP)^12,13^ examines the cognitive operations behind people’s behavioral responses in a social situation. Individuals employ the processed social information together with their previous interactional experiences in order to make sense of and to deal with social situations. Based on previously learned experiences, people subsequently develop practical and functional relational schemes, which consist of the beliefs and expectations about how interpersonal relationships work. These schemes serve as an efficient method to perceive and interact with the world^14^. Furthermore, the efficacy of an individual’s emotional socialization could also affect social information processing. It guides people’s attention towards different aspects of the environment by altering their interpretation of situational cues, and subsequently determining their behaviors^15,16^. This may create biases in social information processing, as individuals may reduce the processing of threatening social information to minimize the discomfort stemmed from their rejected/unaddressed attachment needs in their early years^8,17^.

According to these theories, the importance of early caregiving experiences in affecting social information processing demonstrates the need to explore the relation between parenting practices and its plausible effects on cognitive biases in social information processing. Central to the quality of parenting practices and attachment are the dimensions of care and protection - encompassed in the construct of parental bonding^18^. Parental care refers to the sensitivity of parents towards the child’s needs (emotional, physical, psychological), while parental over-protection refers to the excessive restrictions (emotional, physical, psychological) imposed on the child. The quality of parental bonding influences the development of top-down processes of emotional regulation, which could potentially shape social interactions and physiological responses in individuals. The ability to perceive the social situation as risk-free also has implications for the autonomic nervous system^19^. For instance, levels of paternal care and over-protection received by individuals interacted with genetic characteristics to affect the autonomic response in males towards female distress. Higher levels of paternal care were associated with higher heart rate response towards female cries for individuals with a gene variation that increases sensitivity towards the early caregiving received^20^. Moreover, individuals with overprotective fathers have the tendency to approach others cautiously, while having caring fathers increases a child’s ability to interact with others without inhibition^21^. Other aspects of parental bonding were associated with individual dysfunctional cognitive representations of the world and of the self. For example, maternal care appears to be critical in regulating the self-esteem, extent of trust, proper socialization, and emotional health of the child. In addition, maternal over-protection levels, coupled with low levels of affection, are positively correlated with feelings of being forsaken and experiences of emotional instability^21^.

Generally, the literature points towards higher care and lower over-protection as ideal parenting practices. Parental care increases sensitivity to distressing stimuli and allows individuals to be less restrained in social interactions, while parental over-protection reduces the effectiveness of emotional regulation and the ability to explore social situations. Consequently, adverse parenting practices are also relevant to the manifestation of social anxiety traits in individuals. Parental (maternal and paternal) over-protection and only paternal over-protection are associated with children’s social anxiety and male’s general anxiety levels respectively, while there was no effect of parental warmth on social anxiety levels in individuals^22^.

Social experiences elicit activity in various brain areas such as areas responsible for perception and sensory processing which receive input with social valence (e.g., superior temporal sulcus for social sounds). Activated emotion-related ones (e.g., amygdala) may also be integrated to affect the individual’s response^23^. Additionally, various parts of the prefrontal cortex (PFC) are interlinked to the amygdala and closely involved in the process of social perception and experience. For instance, the orbitofrontal cortex (OFC) is found to be related to weighing the benefits and costs of responding to a social situation. It is also implicated in the valence evaluation of salient social cues^24,25^ by integrating information about environmental context, memory, and emotion^26^. Another relevant area in the PFC is represented by the dorsolateral region, which is linked to areas deputed to motor control (i.e., premotor cortex). This suggests its involvement in responding to environmental stimuli and mechanisms of behavioral regulation and control^26^.

In the context of parental influence, the suppression of emotionally arousing thoughts of negative valence (e.g., separation or loss of a significant other) in anxiously attached individuals has revealed lower activation in the OFC as compared to securely attached individuals. This lower OFC activation suggests that they have a reduced ability in regulating emotions^27^. The existence and implications of a top-down regulation process in individuals’ social experience support the need to study social information processing through examining differential activation of the prefrontal cortex in response to social vocalizations that elicit instinctive responses from individuals. For instance, infant cries act as a biosocial signal to interact and communicate basic needs to adults. Humans are generally “hard-wired” to respond to infant cries by pivoting their attention and motivation towards attending to the needs of the infant^28^. Previous studies have found that infant cries elicit physiological alertness and propel response in adults^29,30^, which is also reflected in neurobiological data. Brain areas relating to the motivation to act and communicate with the infant (e.g., supplementary motor area, superior temporal regions) are activated in mothers upon hearing infant cries, indicating higher sensitivity towards social cues^31^. The left inferior prefrontal cortex involved in the processing and comprehension of sounds is also activated in response to auditory stimuli relating to distressing social signals^31^. Moreover, cries are effective in eliciting negative emotional valence. Hence, they activate brain structures relating to emotional regulation, which drives distress reduction by eliciting intentions to act^32^. An increase in physiological arousal (e.g., higher heart rate) is also found when listening to an infant’s cry, indicating the effect of social cries on emotional and biological arousal^33^. Under typical circumstances, responses towards infant cries include activation of brain areas relating to motivation to act, increase in processing the source of distress, and increase in physiological arousal. Accordingly, cries are a form of salient, social communication of emotions whereby the effects are not limited to merely infant cries. Adult cries, infant cries, and infant laughter are found to increase people’s physiological alertness and propel them into action^30^. This innate response allows for the effective study of individual interaction with social information through examining the corresponding brain activation in reaction to social vocalizations.

Neurobiological and physiological responses to social stimuli are a product of a combination of environmental and genetic factors. Oxytocin receptor gene (OXTr) is widely recognized as a moderator of stress and different aspects of social behaviors^34–36^. Two single nucleotide polymorphisms (SNPs) have been found to be linked to the expression of different social behaviors: rs53576 and rs2254298, both presenting an A to G variation. With regards to rs53576, results in the literature report that the G allele has been associated with more successful social development, such as higher levels of dispositional empathy^37,38^, greater prosocial features^20,37,39,40^, and increased neurocardiac reactivity to social stressors^41^. On the contrary, A/A homozygotes have shown overall poorer social traits^42,43^, lower levels of empathy accuracy^37^, and positive affect^44^, all representing less adaptive social development. Though many results underline the association between specific variations of the genotype with social traits, some inconsistencies still remain^45–47^. For the rs2254298 polymorphisms (G to A variation), G/G homozygotes are shown to be more susceptible to environmental factors compared to the A-carriers, with higher scores in attachment dimensions and separation anxiety^48^. Another study, that has explored the interaction between recalled parental bonding and genetic features, highlights that A-carriers display higher heart rates in responses to social stressors^49^.

Taking all the information together, it appears that modulation of social behavior is a combination of genetic, environmental, and neurobiological factors. Evidence in literature reports results about the involvement of oxytocin receptor gene SNPs in modulating the response to social stressors^50–52^. A NIRS study by Nishitani and colleagues has reported a differential activation of the right inferior anterior prefrontal cortex in G/G homozygotes in response to infant faces^53^. Although it is possible to find studies about the influence of OXTr SNPs on social behavior, the exact role of the specific variations still needs to be fully elucidated. This study aims to shed light on the possible interaction of these factors with measures of neurophysiological arousal (variations of oxygenated haemoglobin in the prefrontal cortex) in response to distressing stimuli in the form of crying vocalizations. Such neurophysiological responses reflect autonomic nervous system activation to psychological stress and resent of both genetic and environmental influences. While genes establish initial patterns of physiological responsiveness, early experiences provide crucial information which may modify and remodel these original patterns, allowing developing individuals to best adapt to their complex and evolving environment.

## Research Question and Hypotheses

Recent studies have begun to explore the effects parental bonding has on the peripheral nervous system response towards social vocalizations. Studies have revealed varied levels of sympathetic nervous system arousal across gender, genetic variation, and recalled early interactions^20,53,54^. However, few studies have investigated the neurobiological consequences/implications of these early parental effects on the development of adaptive social behaviors. Therefore, our study aims to bridge this gap by providing a glimpse into the neurobiological mechanisms underlying how their interaction with parental bonding, perceived in early childhood, could influence emotional and social information processes in social situations. This study would contribute to the current literature by attempting to uncover the rationale underlying the differential neurophysiological reactions and their consequences with varying levels of parental bonding using a social information processing model and genetic features. Since the focus of the research concerns the response to salient social cues, we will focus on the neural activation of the prefrontal cortex, as this region comprehends a set of structures implicated in stimuli appraisal, decisional processes and regulation of social behavior. This research is especially crucial in inferring optimal parental bonding practices, given that their effects are intertwined with the individual’s level of anxiety, adaptiveness towards responding to social vocalizations, and psychopathology. Two hypotheses were hence derived.

- **Hypothesis 1**. Regarding rs53576, we expect A/A homozygotes to show higher neural activation in response to audio stimuli as a signal of greater distress compared to G-carriers. According to evidence in literature, people with this variation tend to present less empathetic responses^37,40^;
- **Hypothesis 2**. G-carriers for OXTr rs53576 and G/G homozygotes of the rs2254298 are expected to show different levels of sensitivity to distressing social stimuli, as the levels of parental care and over-protection increase.

## Results

### Interaction effects of ANOVA analyses

**Figure 1:**
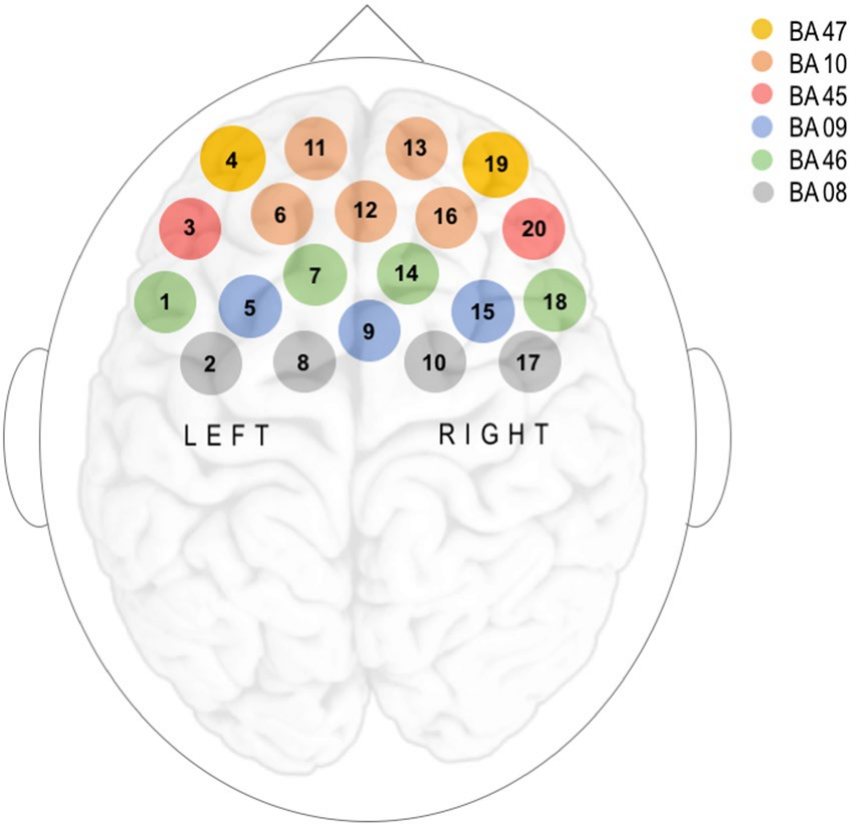
NIRS channels distribution and corresponding Brodmann area covered by each channel in the Prefrontal Cortex.

To have a clearer overview of the results, NIRS channels’ location and coverage in the different areas of the prefrontal cortex are reported in the figure below (see Fig. 1). In our analysis, we employed the Benjamini and Hochberg correction as a false discovery rate (FDR) method to control for multiple comparisons across all the channels^55^. Only significant results after correction are discussed.

#### rs53576

**Figure 2:**
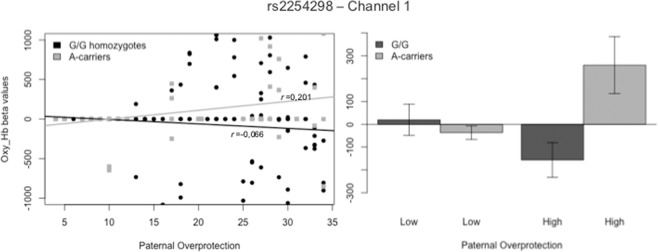
Interaction Effect of rs53576 allelic variations and Paternal Care on Hb_O_ Concentration in Channel 18.

ANOVA analyses of the concentration of Hb_O_ have revealed significant interaction effects between rs53576 and Maternal Care, Paternal Care, Maternal Overprotection, and Paternal Over-protection scores indiscriminately over the entire sound stimuli in five different channels. After correction, a significant interaction result was displayed in Channel 18 between the SNP and Paternal Care (F_Ch18_ (1,245) = 8.158, p = 0.005). Post hoc analysis revealed significant differences between the two SNP variations for low levels of Paternal Care (*t*(133)= −3.242, p = 0.002) and a significant correlation for A/A homozygotes (r_Ch18_A_ = 0.199, p = 0.048), but not for G-carriers (r_Ch18_G_ = −0.144, p = 0.079), highlighting an opposite direction of the effects (see Fig. 2 below). Channel 18 corresponds to BA46R, including the middle frontal gyrus (MFG) and the lateral prefrontal cortex.

#### rs2254298

**Figure 3:**
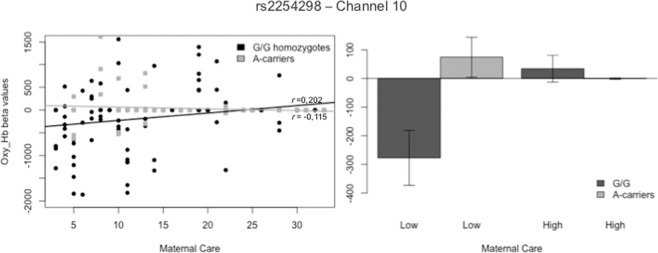
Interaction Effect of rs2254298 allelic variations and Maternal Care on Hb_O_ Concentration in Channel 10.

**Figure 4:**
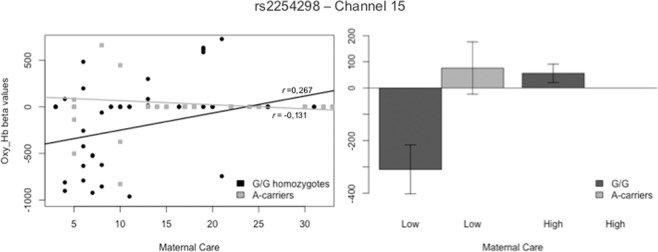
Interaction Effect of rs2254298 allelic variations and Maternal Care on Hb_O_ Concentration in Channel 15. The group “A-carriers High” is not displayed, as beta values are all comprised in a range close to 0 [−0.00000469; 0.00019100].

ANOVA analyses of the concentration of Hb_O_ revealed significant interaction effects between the rs2254298 and Maternal Care, Maternal Overprotection and Paternal Over-protection scores indiscriminately over the entire sound stimuli in five different channels. After Benjamini-Hochberg correction, the interaction between the SNP and Maternal Care was found to exert an effect on channel 10 (F_Ch10_ (1,239) = 7.579, p = 0.006) and channel 15 (F_Ch15_ (1,161) = 7.598, p = 0.007). With regards to channel 10, post hoc analysis revealed significant differences between the two SNP variations for low levels of Maternal Care (*t*(115)= 2.323, p = 0.022). The correlation was significant for G/G homozygotes (r_Ch10_G_ = 0.202, p = 0.009), but not for A-carriers (r_Ch5_A_ = −0.115, p = 0.308) (see Fig. 3). This area refers to the BA08R, which includes the frontal eye field. Likewise, on channel 15, the effect was statistically significant for low levels of Maternal Care and the correlation was significant for the G/G allelic variation (r_Ch15_G_ = 0.265, p = 0.006), but not for the A-carrier individuals (r_Ch15_A_ = −0.131, p = 0.331). This area coincides with the BA09R, corresponding to the left dorsolateral prefrontal cortex (DLPFC). Graphs of the significant results are reported in Fig. 4. Subsequent correlation analysis on the specific nature of the audio stimulus did not find statistically significant results.

**Figure 5:**
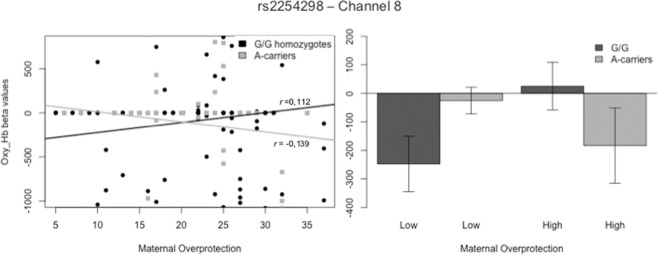
Interaction Effect of rs2254298 allelic variations and Maternal Overprotection on Hb_O_ Concentration in Channel 8.

**Figure 6:**
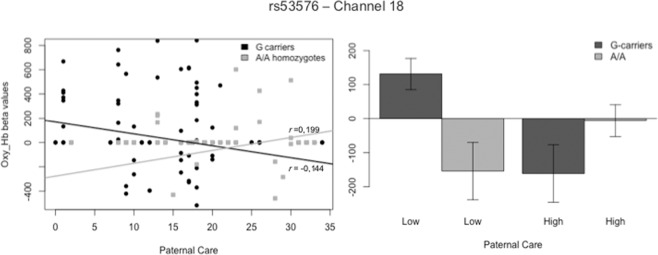
Interaction Effect of rs2254298 allelic variations and Paternal Overprotection on Hb_O_ Concentration in Channel 1.

As for Maternal Overprotection, an interaction effect was found in channel 8 (F_Ch8_ (1,248) = 7.532, p = 0.007). Although graphic representation of result suggested a significant difference between G/G homozygotes and A-carriers for low levels of Maternal Overprotection, post hoc analysis did not confirm these results. Moreover, correlational estimates were not significant, but highlighted an opposite direction of the effects (see Fig. 5. On channel 1, we found an increased prefrontal activity for the interaction effect between rs2254298 and Paternal Overprotection (F_Ch1_ (1,272) = 7.975, p = 0.005). Specifically, significant differences between A-carriers and G/G homozygotes were displayed for high levels of Paternal Overprotection (*t*(133)= 2.958, p = 0.004). In addition, contrary to our hypothesis, a significant correlation for A-carriers (r_Ch1_A_ = 0.201, p = 0.049) was found, but not for the individuals with the G/G variation (r_Ch1_G_ = −0.064, p = 0.376) (see Fig. 6). The area covered by channel 1 overlaps with the BA46L, which includes the middle frontal gyrus and part of the DLPFC.

## Discussion

The purpose of this study is to examine the different responses, in terms of brain activation, towards social stimuli and the nature of neurophysiological mechanisms accompanying the different genetic susceptibility and early childhood experiences. Specifically, we focused on the perceived amount of parental care and over-protection. Moreover, we posited that the neurophysiological responses and activation to social stimuli depend on the allelic variation in two OXTr gene polymorphisms. Overall, a general responsiveness/sensitivity in the perception of emotional vocalizations on parental bonding was found, with both maternal and paternal related subscales exerting an effect on brain activation in both the OXTr gene SNPs.

With regards to **rs53576**, higher levels of Paternal Care were correlated with a greater brain activation in the middle frontal gyrus in A/A homozygotes (Channel 18, BA46R). This area is appointed for functions related to both language and working memory^56^ as well as executive-control processes that inhibit memory retrieval^57,58^. This region has been shown to be involved in top-down attentional processes in recuperate episodes^59^. Hence, a higher activation in A/A homozygotes individuals for higher levels of care, considered to a be a more optimal pattern of parental bonding, represents a less adaptive response to social stressors, confirming **Hypothesis 1**.

Moving to **rs2254298**, Maternal Care resulted in significant brain activation in G/G homozygotes. Higher scores for Maternal Care were correlated with greater levels of Hb_O_ in channel 10 and 15. In channel 10, the frontal eye field is responsible for saccadic eye movements for the purpose of visual field perception and awareness. This suggests that a higher activation may correspond to a readiness to act. Conversely, a decrease in Hb_O_ levels in this area might reflect less propensity to take action in response to social stressful situations. As mentioned, Channel 15 (BA09R) is deputed to inferential reasoning. Individuals with G/G variation presented lower activation of this area in response to social cues for lower levels of Maternal Care, with a higher activity as scores increase. Since this region is related to present vigilance^60^, an increased activation for higher perceived Care might reflect a more efficient response to stressful social stimuli.

Contrary to our second hypothesis, levels of parental overprotection, considered to be a non-optimal pattern of parental bonding, interacted significantly with A-carriers on channel 1, while levels of activation in G/G homozygotes were non correlated. Channel 1 covers the right BA46 which, similar to its left counterpart, regulates inhibitory mechanisms related to memory retrieval^57–59^. This process corresponds to remembering or re-experiencing specific events from the past, as described by Tulving and Markovitsch^61^. Higher inhibitory activity might reflect increased cognitive loading and serve to suppress the occurrence of unpleasant events, hence requiring planning and execution of more adaptive social responses. The observable outcome might result in less favorable social features^37,42,43^. Another plausible explanation could be that G/G homozygotes tend to perceive cries as having less negative emotional valence. As a result, G/G homozygotes may require less cognitive resources for emotion regulation compared to A-carriers.

The results for rs2254298 seem to partially support our **Hypothesis 2**, that receiving better parenting practices could lead to modulation of responsiveness towards social situations. This mechanism could potentially start from the initial stage of social information processing. The larger amount of attention allocated to the listening of the social stimulus characterised by individuals with positive early experiences implicates the extent to which socially related information is being processed and, ultimately, responded to. Individuals whom experienced positive caregiving as characterised by higher levels of parental care and lower parental over-protection also seem to be more motivated to act on or to neutralise the social stimuli.

A noteworthy outcome of the study is that Care-related subscales appear to exert an effect in the right regions of the PFC, while Overprotection-related subscales in the corresponding areas on the left side. This is in line with evidence in literature, reporting the influence of paternal overprotection in the activity of the left DLPFC in young adults^62^. With regards to Care, recalled parental responsibility activates the right anterior insula and inferior frontal gyrus in responses to cries^63^.

The question of nature and nurture in influencing psychological and social processes has been debated over many years, and it has been established that it is insufficient to merely consider the environmental factors. A strength of this study is represented by the effort made in order to enrich the literature on individual sensitivity to social cues by combining genetic features, early life experiences, and objective neurophysiological measures. We utilized an objective measure of brain activation through the brain imaging tool (fNIRS) to examine the impact of perceived parental warmth on individual’s social cognitive processes rather than subjective measures such as self-report. This allows for the control of various biases (e.g. social desirability, the lack of awareness of one’s actual cognitive process/thoughts) which helps in depicting a more accurate picture of the underlying social cognitive processes. Moreover, this study explores parental bonding dimensions related to fathers and mothers, contributing evidence to the importance of considering both parents when investigating the effects of early life interactions on the development of social behavior.

However, there are some limitations: first of all, the sample included a smaller number of individuals carrying the A variation compared to G variation in both polymorphisms. Notably, power is a major concern in gene-environment interaction studies, which requires a very large sample size in order to have enough variability. Albeit the significance of correlations with activation in different areas of the prefrontal cortex relating to social information processing, the correlation coefficient is generally a weak to moderate one. This could be explained by the fact that various parts of the cerebral cortex work together to form a social experience. Moreover, it could also relate to the fact that little variance of the activation in areas of the prefrontal cortex is being explained by early experience of parental bonding. Therefore, more studies are required to examine the effects and control for variables (e.g. other early experiences) that could potentially affect one’s perception of a social behavior. Different physiological measures should also be included in future studies to further complete the picture through better inference and cross reference on the nature of the differential brain activation.

Another limitation of the study involves the fNIRS instrument, as it only measures brain activity up to 4 cm of the cerebral cortex, which could pose limitations for examining social behaviors. Although areas contained in the prefrontal cortex have been proved to be linked to subcortical structures, social behaviors typically involves emotions, which are processed in areas such as the nucleus accumbens and the amygdala. Investigations on these areas would allow a better understanding of the nature of interaction and processes involved in perception of social situations.

## Conclusion

The general idea that parental bonding practices influence social information processing when interacting with environmental susceptibility-related variation of two main SNPs of oxytocin receptor gene was supported by our results. Receiving better parenting practices could, indeed, lead to an increased activity in prefrontal cortex areas relating to responsiveness towards social situations, and to a decreased activity in response inhibition areas. This pattern of brain activity could facilitate better processing and understanding of the communication of social needs. Moreover, it also increases brain activation in areas relating to one’s motivation to act and respond to the needs of social others. Generally, the influence of early parental care and over-protection on the prefrontal activation are more pronounced in G/G homozygotes than in A-carriers, for rs2254298. That is, changes in the levels of parental over-protection and care values could lead to significant changes in the individual with G/G variant brain responses to social vocalizations, while there is a relatively constant brain responses in A-carriers. With regards to the rs53576, the effects were observed in A/A homozygotes, showing that the direction of the correlation tends to be more related to the social cue than to parental care or over-protection. Our study also highlights the importance of a father’s contribution towards parenting of the child. While a mother’s caregiving behaviors are often emphasized and put in the spotlight, this study illustrates the potential effect paternal caregiving behaviors have on a child, especially with regard to the care. Therefore, parenting, as an initial learning of how to communicate effectively with others, seems to require the involvement of both parents. Finally, this study sheds light on how early parental care and over-protection can affect the process of perceiving social information, supplementing an account to understand the neurological mechanisms behind the negative impact that poor parenting practices have on the outcomes of the child.

## Methods

One hundred thirty-four participants consisting of 84 females (M_age_ = 21,16, SD = 1.92) and 50 males (M_age_ = 21.16, SD = 1.91) were recruited via the undergraduate research participation pool from the Nanyang Technological University of Singapore (NTU). Having children was set as an exclusion criterion for recruitment. Participants were compensated with 3 credits for their time spent (1 credit/0.5 hour), which contributes to a psychology module’s course assessment. The study was approved by the ethics committee of NTU Institutional Review Board. Participants’ informed consent was obtained before the starting of the study. All methods were performed in accordance with the relevant guidelines and regulations and the study was approved by the Institutional Review Board of Nanyang Technological University of Singapore. All data are available at this URL: 10.21979/N9/VXF4XB.

### Perceived parental bonding

Parental Bonding Instrument (PBI)^18^ was utilized to measure individual’s perceived parent-child attachment in the initial 16 years of their life (see Appendix A for the questionnaires). Two dimensions namely “care” and “over-protection” were explored. “Care” examines the extent to which affection and sensitive parenting were perceived to be provided by both parents (e.g. “seemed emotionally cold to me”, “spoke to me in a warm and friendly voice”). Individuals with “low care” scores regard parents as rather distant, unsympathetic and unresponsive; while individuals scoring “high care” have a caring, sympathetic, and affectionate experience of parental bonding. “Over-protection” examines the extent to which individuals perceive parents to implement excessive control and/or to impede their growth towards independence (e.g. “invaded my privacy”, “felt I could not look after myself unless she/he was around”). Participants responded to 12 “Care” and 13 “Over-protection” items on a scale ranging from “very unlike”, “moderately unlike”, “moderately like”, to “very like”. To explore the quality of bonding with both parents, PBI comprises of two identical forms (25 items each) for father and mother, respectively. PBI was proven to have stability over time in studies with test-retest after a period of 20 years, and was relatively unaffected by mood changes^64–66^. PBI was also found to be closely related to the Egna Minnen Betrsffande Uppfostran (EMBU) questionnaire, which attempts to measure similar constructs of warmth and over-protection^67^. Here, the Cronbach’s alpha of the “care” dimension for mother is *α* = 0.94, and for father is *α* = 0.95, implying excellent internal consistency. For the “over-protection” dimension, the Cronbach’s alpha value for mother and for father are *α* = 0.87 and *α* = 0.90 respectively, thus suggesting acceptable to good internal consistency.

### Auditory stimulus

Three different types of non-linguistic vocalizations were utilized in this study, namely: women cries (WC), infant cries (IC), and cat cries (CC). Infant vocalizations were obtained at the age of 3 months from birth. CC were included to take into account the effect of vocalizations across species. The sounds of IC, and WC (sampling rates 44.1 kHz/32 bit) were acquired from the Oxford Vocal Sounds database^30^. CC were obtained from selected clips from YouTube (www.youtube.com), and 15 seconds snippets of the clips were obtained using the online Audio Trimmer (http://audiotrimmer.com). Subsequently, Audacity 2.0.4 (http://sourceforge.net/projects/audacity/files/audacity/2.0.4/) was used to minimize the background noise and ensure the compatibility of volume to other sounds. These emotionally salient sounds were chosen with the intention of mimicking social situations found to instinctively elicit response in individuals. Therefore, the ratio of 15 seconds of auditory stimuli (infant cry, women cry, cat cry) was selected: 10 seconds of inter-stimulus interval were utilized in this study to elicit maximal emotional valence of a social situation (see^68^) in an experimental setting. The sound stimulus consisted of 10 tracks from each type of vocalization, making 30 tracks. NIRStim software was used to construct the approximately 15 minutes-long sound stimulus (inclusive of inter-stimulus intervals), and to randomize the order of presentation of the auditory stimulus to the participants.

### Functional near-infrared spectroscopy (fNIRS)

A fNIRS (NIRSport, NIRx Medical Technologies LLC) was employed in this study to acquire data on the relative changes in concentration levels of oxygenated haemoglobin (Hb_O_) and deoxygenated haemoglobin (Hb_R_) in the prefrontal cortex. In the presence of activation of a cerebral area the higher metabolic demands would require more Hb_O_ to support its activity, thus resulting in greater concentration of Hb_O_ to Hb_R_, which is captured by the equipment. As hemodynamic responses (cerebral blood flow) correspond closely to neuronal activity, it is possible to infer the level of brain activity via measuring the concentration levels of the Hb_O_ and Hb_R_ in a cortical area. The instrument scans with a rate of 7.81 Hz and emits light with source wavelengths of 760 nm and 850 nm, specific to the absorption spectrum required for detection of Hb_O_ and Hb_R_ respectively. An 8 × 7 source-detector montage was employed, and the prefrontal cortex activity was registered using a 20 channels configuration. Only the Hb_O_ values were reported in the subsequent analyses as they have a higher correspondence with cerebral haemodynamic responses than Hb_R_ values, facilitating a higher accuracy during the inference of brain activity^69^.

### Genetic collection and SNP genotyping

Genomic DNA was collected by asking participants to scrub the inner part of both cheeks for at least 30 seconds each side using a buccal swab. Samples were placed and left to dry for 48 hours in a 1.5 ml tube in a dry place. DNA was extracted from buccal swabs following the procedure reported by Bonassi and colleagues^70^, using Oragene DNA purifying reagent. DNA concentrations were measured using spectroscopy technology (NanoDrop Technologies, USA). Polymerase chain reaction (PCR) procedure magnified the concentrations for the OXTr gene rs53576 region target with the primers 5-GCC CAC CAT GCT CTC CAC ATC-3 and 5-GCT GGA CTC AGG AGG AAT AGG GAC-3. A PCR reaction of 20 ll was conducted, including 1.5 ll of genomic DNA from the test sample, PCR buffer, 1 mM each of the forward and reverse primers, 10 mM deoxyribonucleotides, KapaTaq polymerase, and 50 mM MgCl2. The PCR procedure consisted of a first stage of denaturation at 95 °C (15 min), following 35 cycles at 94 °C (30 s), 60 °C(60 s), 72 °C (60 s) and a final protraction at 72 °C (10 min). PCR reactions were genotyped with an ABI 3730xl Genetic Analyzer (Applied Biosystems Inc.) and standardized with GeneScan 600 LIZ (Applied Biosystems, Inc.) for each sample. A similar procedure was applied for the OXTr gene rs2254298 region target, except that different forward and reverse primers were 5-TGA AAG CAG AGG TTG TGT GGA CAG G-3 and 5-AAC GCC CAC CCC AGT TTC TTC-3 instead. Genotyping for allelic variants of rs2254298 and rs53576 SNPs was performed using the TaqMan genotyping platform in accordance with the manufacturer’s protocol. With regards to rs53576 region, participants having at least one G allele (G/G homozygotes or A/G) were classified into a single G-carriers group. The averaged distribution of the different genotypes in the Asiatic population is 45–65% for A/A homozygotes and 35–55% for G-carriers (1000 Genomes project, BioSamples: SAMN07486027-SAMN07486024, dbSNP, 2017), whereas the distribution in our sample was 38.24% for A/A homozygous and 61.76% for G-carriers. In detail, genotype frequencies were as follows: A/A = 39 (38.24%), A/G = 49 (48.04%), G/G = 14 (13.73%). This genotype distribution follows the Hardy-Weinberg Equilibrium (*X*^2^(1) = 0.05; *p*-value = 0.82). Participants’ gender did not significantly differ between the two groups A/A vs G (*X*^2^(1)= 1.2938, p-value = 0.2553). For rs2254298 region, participants having at least one A allele (A/A homozygotes or G/A) were classified into a single A-carriers group. The average distribution of the different genotypes in the Asiatic population is 65–90% for G/G homozygotes and 10–35% for A-carriers (1000 Genomes project, BioSamples: SAMN07486027-SAMN07486024, dbSNP, (Short Genetic Variations), 2017), while the distribution in our sample was 64.71% for G/G homozygous and 35.29% for A-carriers, composed as follows: A/A = 9 (8.82%), G/A = 66 (26.47%), G/G = 66 (64.71%). Although the Hardy-Weinberg Equilibrium is not met, probably due to the moderate size of the sample (*X*^2^(1) = 5.40; *p*-value = 0.02), the allelic variation distribution is adequate to proceed with analyses. Participants’ gender did not exert any effect in for both SNPs (for rs53576 *X*^2^(1) = 1.203; *p*-value = 0.273; for rs2254298 *X*^2^(1) = 2.279; *p*-value = 0.131).

### Design and procedure

This study employed a 2 (Care: Mother, Father) × 2 (Over-protection: Mother, Father) × 3 (Sounds: Infant Cry, Cat Cry, Woman Cry) × 2 (Genetics: A-variant vs G-variant) factorial, quasi-experimental design. The study comprises of two experimental sessions. The first one was conducted on a Qualtrics online platform. Upon consenting to participate in the study, participants were asked to complete the PBI questionnaire online. Demographic variables relevant for the study (e.g. gender, age, and ethnicity) were included as starting questions in the online questionnaire. The second experimental session took place in the Social and Affective Neuroscience Laboratory. Upon acquiring informed consent, the NIRS cap was mounted on the participants with time factored in to calibrate, inspect, and adjust the signal quality for the NIRS and participants were reminded to minimize motion. All participants were asked to listen to a series of randomized sound stimulus (30 tracks) lasting approximately 15 minutes while NIRS recordings were made. Initial 30 seconds of the experiment consisted of information about the nature of the sounds that they would be exposed to during the course of experiment. Participants were instructed to fixate their gaze on the white cross (“+”) that would be presented on the centre of the black screen throughout the entire stimulus presentation, attempting to control for eye movements. After the instructions, a 15 seconds soundtrack (randomized) was presented, followed by a 10 seconds inter-stimulus interval of recovery and the next 15 seconds trial, until all 30 soundtracks were presented. Before debriefing, a sample of the participant’s buccal mucosa was collected using a buccal swab and then sent to the laboratory for DNA-related information extraction.

### NIRS data acquisition and pre-processing

Preceding the analyses of the NIRS data, NirsLAB v.2016 software was utilized to pre-process the data. Initially, the quality of the signals recorded on each channel was inspected via the labelling as “bad” the channels containing exceptional amount of background noise (Gain > 8, Coefficient of Variation (CV%) > 7.5, NaN). Only “good” signals were considered for the subsequent pre-processing stages. Following, markers for the onset of each stimulus were set, labeling the timing according to the type of condition. Discontinuities and spikes artifacts were removed manually and signals were cleaned from eventual remaining irregularities (i.e., due to slow signals and/or alterations in the baseline shift) by applying a band-pass filter [0.01–0.2 Hz]. Haemodynamic states were subsequently computed for each channel, and the Beer-Lambert law was used to convert processed signals in variations of oxygenated (Hb_O_) and deoxygenated haemoglobin (Hb_R_) concentrations. NIRSLab v.2016 software was subsequently utilized for statistical parameter analysis. Haemodynamic Response Function (HRF) was selected to improve the synchronization between haemodynamic and neural activities. Next, a Discrete Cosine Transformation (DCT) temporal parameter was applied, with a high-pass period cut-off of 128 sec (0.008 Hz), to reduce the possible residual portion of data variance not related to the experimental hemodynamic states. Afterwards a Gaussian pre-coloring filter was selected and the Full-Width at Half-Maximum (FWHM) was set at 4 seconds, to maximize the accuracy of the statistical inferences of the General Linear Model (GLM) computations, which occurred as the final stage of the pre-processing procedure. Specifically, within-session and within-subject General Linear Model (GLM) analysis of fNIRS hemodynamic-state time series were obtained for each participant, based on the (Hb_O_) processed signals. GLMs evaluated the relationships between channel responses and the temporal sequence previously set, resulting in an output of a matrix containing the beta-coefficients for each time frame condition, for each of the 20 channels, and then aggregated in average beta-coefficients for each of the 3 audio conditions. GLMs average beta-coefficients from 134 participants, consisting of 84 females (M_age_ = 21.16, SD = 1.92) and 50 males (M_age_ = 21.16, SD = 1.91), were included in the first version of the database. Once results on OXTr SNPs were received from the laboratory, the final dataset was built, comprising a total of 102 subjects: for rs53576, the sample consisted of 39 A/A homozygotes (14 Male; 25 Female) and 63 G-carriers homozygotes (21 Male; 42 Female); as for rs2254298, the sample consisted of 36 A-carriers (14 Male; 22 Female) and 66 G/G homozygotes (21 Male; 45 Female).

### Data analysis

Preliminary data analysis highlighted that sex of the participants did not have any effect on the neural responses to audio stimuli. For this reason, sex is not taken into account in the analysis nor in the discussion of our results. As for ethnicity, the total sample consisted of 81 Chinese people, 11 Malaysians, 5 Indians, 2 Caucasians (unspecified), 1 Filipino and 2 Africans, as self-reported in the demographic section of the questionnaire. There were no significant differences among groups, hence we analysed the sample as a whole East-Asian population, as the “1000 Genomes project” does, considering their frequency of alleles variation as a reference (see the 1000 Genomes Project database http://www.ensembl.org/Homo_sapiens/Info/Index) Moreover, ethnicity did not exert any effect for OXTr rs53576 (*X*^2^ = 2.416, p = 0.789) nor for rs2254298 (*X*^2^ = 2.962, p = 0.706). Utilising the R-studio software, 20 six way between-subject ANOVAs were conducted, one for each of the NIRS channels. Before proceeding with the analyses, the database was screened and cleaned for missing values or values equal to 0 in all the channels in order to keep only the reliable ones. Missing values in NIRS channels might be due to an interruption of the signal while recording. Variables were then checked for skewness and kurtosis, showing an acceptable normal distribution. Six independent variables, namely (i) genetic variations, (ii) maternal over-protection, (iii) paternal over-protection, (iv) maternal care, (v) paternal care, and (vi) different types of social vocalizations were considered in the analyses. Subsequently, t-tests for interaction effect for categorical variables (e.g. allelic variation, and “High” vs. “Low” levels of parental care and overprotection) were conducted for the channels with significance, which passed the Benjamini-Hochberg correction.
